# Stimulation of GABA Receptors in the Lateral Septum Rapidly Elicits Food Intake and Mediates Natural Feeding

**DOI:** 10.3390/brainsci12070848

**Published:** 2022-06-28

**Authors:** Ivett Gabriella, Andy Tseng, Kevin O. Sanchez, Himani Shah, Billy Glenn Stanley

**Affiliations:** 1 Department of Psychology, University of California Riverside, Riverside, CA 92521, USA; stanley@ucr.edu; 2 Graduate Program in Neuroscience, University of California Riverside, Riverside, CA 92521, USA; atsen002@ucr.edu; 3 Department of Molecular Cell and Systems Biology, University of California Riverside, Riverside, CA 92521, USA; kevinosanchez928@gmail.com (K.O.S.); hshah004@ucr.edu (H.S.)

**Keywords:** eating, feeding, lateral septum, gamma-aminobutyric acid, GABA receptors, rat model, central injections

## Abstract

The increasing prevalence of obesity and eating disorders makes identifying neural substrates controlling eating and regulating body weight a priority. Recent studies have highlighted the role of the lateral septum (LS) in eating control mechanisms. The current study explored the roles of gamma-aminobutyric acid (GABA) receptors within the LS in the control of food intake. Experiments with a rat model (n ≥ 11/group) showed that LS microinjection of the GABA_A_ receptor agonist, muscimol, and the GABA_B_ receptor agonist, baclofen hydrochloride (baclofen), elicited intense, dose-dependent feeding. In contrast, LS pretreatment with the GABA_A_ receptor antagonist, picrotoxin, markedly reduced the muscimol-elicited feeding, and pretreatment injections with the GABA_B_ receptor antagonist, 2-hydroxysaclofen (2-OH saclofen), reduced the baclofen evoked response. Next, we showed that picrotoxin injection at the beginning of the dark phase of the light-dark cycle—when rats show a burst of spontaneous eating—reduced naturally occurring feeding, whereas 2-OH saclofen was ineffective. These results indicate that the activation of LS GABA_A_ and GABA_B_ receptors strongly stimulates feeding and suggests potential roles in feeding control neurocircuitry. In particular, our evidence indicates that endogenous LS GABA and GABA_A_ receptors may be involved in mediating naturally occurring nocturnal feeding.

## 1. Introduction

Disturbances in eating may lead to physical and mental health challenges. For example, the prevalence of obesity among US adults continues to increase, currently exceeding 42% [1]. When these disturbances effect psychosocial functioning, feeding and eating disorders may develop, such as anorexia nervosa, bulimia nervosa, or binge eating disorder [2]. The estimates of the life time prevalence of eating disorders reached 0.8% for anorexia nervosa, 0.28% for bulimia nervosa, and 0.85% for binge eating disorder [3]. These high levels and significant increases in rates of obesity [1] and eating disorders [3] highlight the importance of understanding the brain mechanisms controlling feeding behavior and body weight regulation.

Recent research suggests that the lateral septum (LS) is a key relay in feeding control neurocircuitry [4,5,6]. The LS has bidirectional projections to the hippocampus, the lateral and medial hypothalamus, and the amygdala [7], which mediate its involvement in positive reinforcement [8], drug reward [9] and feeding behavior [10,11]. Several of the brain regions the LS connects to have been examined and implicated in feeding, such as the lateral hypothalamus ([12,13]) and the nucleus accumbens ([14,15]). In addition, Azevedo et al. [16] reported that neurotensin-expressing neurons in the LS responded to stress, and their activation led to decreased food intake. Furthermore, Terril et al. [17] found that food intake increased overnight when glucagon-like peptide 1 receptors were blocked in the dorsal lateral septum, and the injection of morphine into the LS elicited a delayed feeding response [18,19]. These studies suggest complex roles for LS neurons and their connections in controlling feeding behavior.

Gamma-aminobutyric acid (GABA) is the dominant inhibitory neurotransmitter in the central nervous system [20] via its actions on GABA_A_ and GABA_B_ receptors. Since the predominant type of neuron in the LS is the medium-sized GABAergic spiny projection neuron [21], it is unsurprising that LS GABA receptors are involved in inhibitory feeding mechanisms. In particular, chemogenetic and optogenetic stimulation of GABAergic projections from the septum to the lateral hypothalamus suppressed food intake [13] showing the inhibitory role of LS in feeding. Moreover, LS GABAergic neurons not only synapse on surrounding brain regions, but they also synapse on and inhibit neighboring LS neurons [7], thus producing disinhibition. In alignment with this, several studies have shown that activating projections to the LS from other brain regions suppresses feeding. Photostimulation of glutamatergic projections from the paraventricular hypothalamus to the ventral LS reduced feeding [22], as did optogenetic activation of glutamatergic projections from the ventral hippocampus [23]. Similarly, Azevedo et al. [16] showed that chemogenetic activation of hippocampal dopamine 2 receptor neurons projecting to the septum decreased food intake. With their myriad projections and multifaceted actions, these LS GABAergic neurons may be central to an inhibitory circuit underpinning feeding.

However, less research has investigated the role of LS GABA_A_ and GABA_B_ receptors. Mitra and colleagues [24] showed that activating either GABA_A_ or GABA_B_ receptors in the LS increased sucrose intake, with activation of GABA_A_ receptors being more effective than GABA_B_ receptors. Moreover, Calderwood et al. [18] found that muscimol, a GABA_A_ receptor agonist, elicited feeding in the LS much more rapidly than the mu-opioid receptor agonists. Specifically, the onset of feeding occurred within 30 min of muscimol injection [25]. In addition, the injection of a GABA_A_ agonist resulted in a greater feeding response within the LS than in any other area surrounding the LS—indicating a site specific activation [26]. These studies underscore the need to investigate the function of both GABA_A_ and GABA_B_ receptors for our complete understanding of the LS’s contribution to eating behavior.

The objective of the current study was to clarify the role of LS GABA_A_ and GABA_B_ receptors in feeding. The first aim was to determine whether activation of LS GABA_A_ or GABA_B_ receptors with agonists would elicit feeding that could be suppressed by pre-treatment with antagonists. We showed that agonists of GABA receptors would produce a feeding response while pre-treatment with antagonists would suppress it. The study’s second aim was to determine whether endogenous LS GABA naturally activates GABA receptors in the LS to elicit feeding. Because rats feeding activity exhibits a peak during the beginning of the dark phase [27], this period provided an opportunity to address this question. Results showed that GABA receptor antagonists would suppress food intake at the onset of the dark phase when rats eat their largest meal, revealing roles for endogenous LS GABA and GABA receptors in this behavior.

## 2. Materials and Methods

### 2.1. Animals

The subjects of the experiment were adult male Sprague--Dawley rats (Charles River Laboratories, Wilmington, MA, USA) weighing 350 to 400 g at the time of the surgery. Rats (n = 75) were housed individually in a temperature-controlled vivarium on a 12:12-h light-dark cycle. All experiments were approved by the Institutional Animal Care and Use Committee at the University of California, Riverside, and met all expectations provided by the Guide for Use and Care of Laboratory Animals [28]. In addition, the procedures complied with the ARRIVE (Animal Research: Reporting of In Vivo Experiments; [29]) guidelines.

### 2.2. Surgical Procedures

We performed surgeries to implant cannulas into the LS permanently. Animals were injected before surgery with 0.4 mL of atropine sulfate (0.54 mg/mL) to reduce salivation and bronchial secretions. Then, they were anesthetized by intraperitoneal injection of a mixture of Ketamine HCl (80 mg/mL) and Xylazine HCl (12 mg/mL) at 1 mL/kg of body weight. A stainless-steel guide cannula (26 gauge, 18 mm long) was stereotaxically implanted unilaterally into the LS 1 mm above the target site. The coordinates used were: 0.0 mm anterior to Bregma, 0.9 mm lateral to the midsagittal sinus, and 4.5 mm ventral to the surface of the skull, with the incisor bar at −3.3 mm. Cannulas were held in place by four stainless-steel skull screws and dental acrylic. A plastic guard was embedded into the dental acrylic to protect the cannulas, which were sealed with a removable 33-gauge stainless-steel obturator. Rats were given at least seven days to recover from surgery, during which they were handled and mock-injected multiple times to habituate them to the injection procedures.

### 2.3. Specific Experiments

Experiments 1–4 examined Aim 1. In these four experiments, injections were separated by at least 48 h and given within the first 3 h of the light phase of the light-dark cycle, when rats rarely eat spontaneously [30]. Subjects were injected with aCSF on the first test day. In subsequent days, rats were injected in counterbalanced order with three different doses of muscimol (Experiment 1) or baclofen (Experiment 2) as shown in Figure S1). In Experiment 3, rats were injected in counterbalanced order with one of the three doses of picrotoxin (0.05, 0.1, and 0.2 μg/0.3 μL), followed within approximately 30 s by an injection of muscimol (0.2 μg/0.3 μL). In Experiment 4, rats were injected in counterbalanced order with one of three doses of 2-OH saclofen (0.5, 1.25, 2.5 μg/0.3 μL) followed immediately by baclofen (1.125 μg/0.3 μL). This sequence allowed the antagonist to suppress the action of GABA receptors before the injection of the agonists. Because the rats received two sequential injections, they were allowed three days of rest between tests (Figure S1).

Aim 2 was tested in Experiments 5 and 6. These experiments had matching injection schedules, with all of the injections performed within the first 30 min of the onset of the dark phase, and the tests were separated by 48 h. Rats received an aCSF injection on the first and fifth test days. On the second, third, and fourth test days, the rats were injected with picrotoxin (0.1 μg/0.3 μL) in Experiment 5 and 2-OH saclofen (1.25 μg/0.3 μL) in Experiment 6 (Figure S1).

### 2.4. Test Procedures

Rats had ad libitum access to Purina rat chow and water until three days before the first injections. Then, their regular rat chow was replaced with a highly palatable sweet milk mash diet available ad libitum. The mash food consisted of a mixture of Purina rat chow powder (500 g), sugar (400 g), and condensed milk (354 mL, Carnation). Its moist texture limits the amount of water needed for chewing and digesting, minimizing meal-associated drinking. Rats were additionally given two Purina rat chow pellets between injection days as enrichment. To ensure satiation, rats were fed fresh mash food 1 h before injections in Experiments 1–4. Injections were through an injector extending 1 mm past the cannula onto the LS, with a 10-sec pause before removing injectors. Food and water intake were measured 1, 2, 4, and 24 h after injections.

### 2.5. Drugs

In Experiment 1, the GABA_A_ receptor agonist muscimol was injected into the LS at doses (0.1, 0.2, 0.3 μg/0.3 μL) based on Calderwood et al. [18]. Muscimol is a full agonist and selectively binds to ionotropic GABA_A_ receptors with high affinity [31,32]. In Experiment 2, the high-affinity GABA_B_ receptor agonist, baclofen, was injected into the LS. Doses (0.45, 1.125, 2.25 μg/0.3 μL) were based on Mitra et al. [24]. Experiments 3 and 5 used picrotoxin, a GABA_A_ receptor antagonist, which has a rapid onset of action and non-competitively binds near or on chloride channels [31]. Previous studies have used picrotoxin in the LS at a wide range of doses [33,34,35,36]; therefore, three doses (0.05, 0.1, and 0.2 μg/0.3 μL) were chosen in the sub-seizure middle range of those used in previous studies. The competitive GABA_B_ antagonist 2-OH saclofen [37,38] was used in Experiments 4 and 6. The active enantiomer, 2-(S)-(+)-2-hydroxy-saclofen (doses: 0.5, 1.25, 2.5 μg/0.3 μL), was selected to yield a small, medium, and large dose. All drugs were purchased from Tocris Bioscience (Minneapolis, MN, USA) and dissolved in artificial cerebral spinal fluid (aCSF; 147 mM Na^+^, 154 mM Cl^−^, 3 mM K^+^, 1.2 mM Ca^2+^, and 0.09 mM Mg^2+^), except 2-OH saclofen, which was dissolved in distilled water for improved solubility. The injection volume was 0.3 μL in all cases.

### 2.6. Histological Procedures

After behavioral tests were complete, rats were injected with Chicago Sky Blue 6B (Alfa Aesar, Thermo Fisher Scientific, Waltham, MA, USA) stain to mark the injection site. They were immediately euthanized with an overdose of sodium pentobarbital (Fatal Plus, Vortech Pharmaceuticals, LTD, Dearborn, MI, USA) and transcardially perfused with formaldehyde (10%). Histological procedures included sectioning 100 μm coronal brain slices on a freezing microtome and Nissle staining with cresyl violet to verify the injection site. Injection sites were matched to the coordinates by Paxinos and Watson [39]. Rats with injection sites outside the LS target area were excluded from the study.

### 2.7. Statistical Analysis

The dependent variables were the measured food and water intake in all analyses. Two-way repeated-measures ANOVA (Analysis of Variance) was used to assess food and water intake at 1, 2, and 4 h. The 24 h food intakes were evaluated by one-way repeated measures ANOVA. For these ANOVA tests, sphericity assumptions were checked by Mauchly’s test [40], and Greenhouse–Geisser [41] correction was applied if the data did not meet the assumptions. Significant ANOVA tests were followed by paired-samples *t*-tests between each pair of variables at matched post-injection times. To correct for Type II error, Bonferroni correction [42] was applied to all p-values.

To evaluate if the rats in our samples exhibited typical eating behavior, the food intakes 1, 2, and 4 h after aCSF injection in each experiment were compared to spontaneous feeding at matched times during the light and the dark phases in two randomly selected groups of naive rats (n = 25 × 2). Food intake results of the naive rats are shown in Table S1. There were no statistically significant differences between the spontaneous food intakes and those following aCSF injections in any of the experimental groups. Rats that died before the first aCSF injections were excluded from the analysis. Additionally, three rats completed all but the last test. This resulted in 24 missing values, which were inspected with Little’s Missing Completely at Random test, and expectation-maximization values were used to replace them.

All statistical analysis was performed using IBM Statistical Product and Service Solutions (SPSS) 27 software. Data are expressed as means ± 1 standard error of the mean (SEM). Effect sizes are indicated as Partial Eta Squared (${\eta_{p}}^{2}$). The “n” numbers indicate the number of animals used for each experiment. Data sets and codes of statistical analysis are available on the Open Science Framework website [43].

## 3. Results

### 3.1. Histology

**Figure 1:**
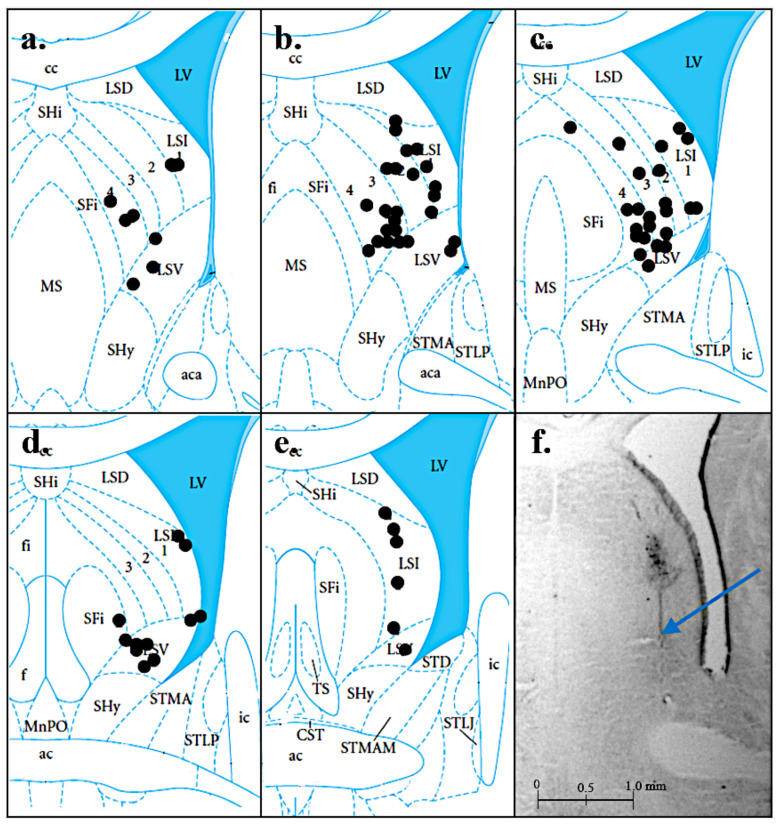
Histology results. Black circles indicate the injection sites in the lateral septum (LS). Panels (**a**–**e**) show different rat brain sections at interaural coordinates 9.2, 9.1, 9.0, 8.9, and 8.7 (respectively) based on Paxinos and Watson (2013) [39]. Panel (**f**) shows a histological image of a cresyl violet stained rat brain section showing a representative injection site (marked by the arrow) at 9.0 interaural coordinates. Brain sections are reprinted from The Rat Brain in Stereotaxic Coordinates: Hard Cover Edition (7th), by G. Paxinos, and C. Watson, 2013. Elsevier. by Academic Press.

We performed histology after each experiment to verify the location of the injections. Injection sites were verified in 72 of the 75 rats. Due to damage or loss of brain tissue during the experiments, injection sites could not be verified for three rats. As shown in Figure 1, all but three injection sites were localized in the LS, ranging from 8.7 to 9.2 mm anterior to the interaural line, including a substantial number extending ventrally into the ventral lateral septum.

### 3.2. Experiment 1: Activation of LS GABA_A_ Receptors Elicits Feeding

**Figure 2:**
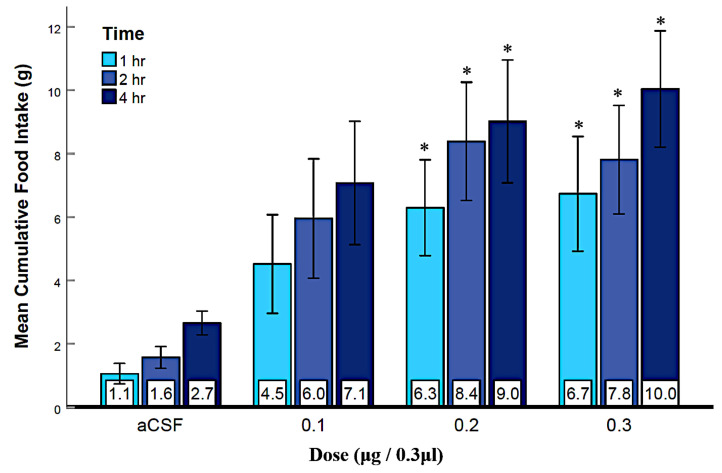
Muscimol injection into the LS during the light phase rapidly elicited eating. Error bars represent mean ± SEM; n = 15; mean values are shown at the bottom of each bar. * indicates significant differences at *p* < 0.05 compared to the aCSF group at matched times.

To investigate a feeding stimulatory role for GABA_A_ receptors in the LS, central injections of the GABA_A_ receptor agonist, muscimol, were performed. As shown in Figure 2, muscimol rapidly and consistently elicited food intake in a dose-dependent fashion. Two-way repeated-measures ANOVA (n = 15) showed that both dose (F_(3, 13)_ = 4.1, *p* = 0.01, ${\eta_{p}}^{2}$ = 0.23) and time (F_(1.13, 13)_ = 13.2, *p* = 0.002, ${\eta_{p}}^{2}$ = 0.48) variables were significant. The interaction of dose and time was not significant. Post hoc analysis revealed that the 0.2 and 0.3 μg doses produced significantly more food intake than the aCSF group. Each of the three time points was also significant from each other (Figure 2). Muscimol injection did not affect water intake (Table S2), suggesting that muscimol’s effect was specific to eating. Average daily food intake at 24 h was similar to the food intake in the aCSF condition for all three doses of muscimol. This suggests that the feeding response elicited by GABA_A_ receptor activation is short-term, with the initial overeating being compensated for by less eating afterward. These results provide support a role for LS GABA_A_ receptors in feeding stimulation.

### 3.3. Experiment 2: Activation of LS GABA_B_ Receptors Elicits Feeding

**Figure 3:**
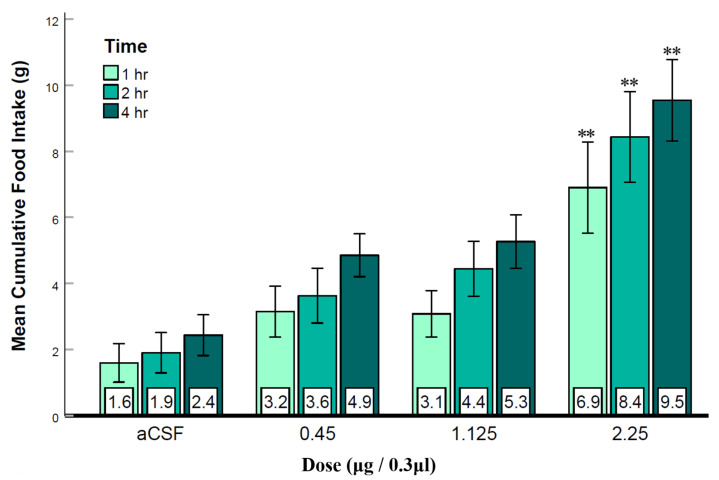
Baclofen injection into the LS during the light phase rapidly elicited eating. Error bars: ±SEM; n = 13; mean values are shown at the bottom of the bar. ** indicates significant differences from the aCSF group at *p* < 0.01.

In this experiment, LS injection of the GABA_B_ receptor agonist baclofen was used to examine whether LS GABA_B_ receptor activation was sufficient to elicit feeding. As shown in Figure 3, baclofen injected into the LS rapidly and consistently elicited feeding in rats. Repeated measures ANOVA (n = 13) revealed significant main effects for both dose (F_(3, 11)_ = 10.06, *p* < 0.001, ${\eta_{p}}^{2}$ = 0.46) and time (F_(1.38, 11)_ = 24.65, *p* < 0.001, ${\eta_{p}}^{2}$ = 0.67). The interaction of dose and time was not significant. Post hoc analysis showed that only the highest dose of baclofen was significantly different from the aCSF and the smallest dose of baclofen, indicating a need for relatively large doses for sufficient GABA_B_ receptors to be activated. The three different time points were also significant from each other. These results showed that the highest dose of baclofen elicited a strong feeding response within one-hour post injection (Figure 3). The 24 h measurements did not reveal statistically significant differences (figure not shown), and water intake was also not affected (Table S3). This suggests that baclofen’s effects lasted only a few hours after injection, but they were specific to feeding. These results convey that, in addition to GABA_A_ receptors, LS GABA_B_ receptor activation alone may also be sufficient to elicit feeding.

### 3.4. Experiment 3: Suppression of LS GABA_A_ Receptors Reduced Agonist-Elicited Feeding

**Figure 4:**
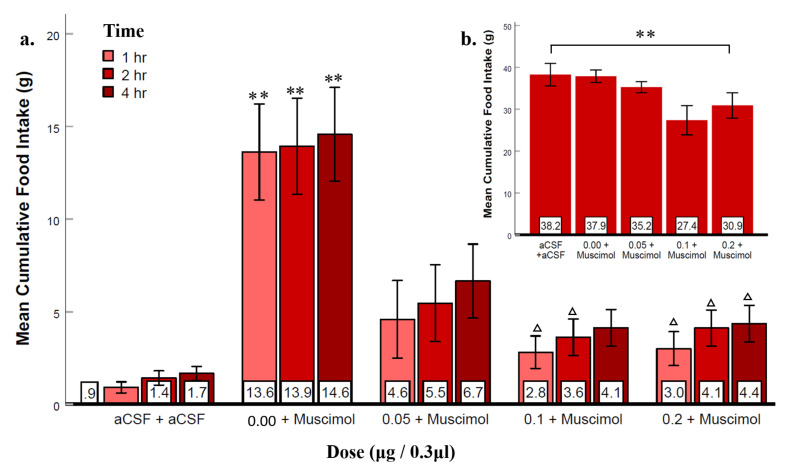
Pretreatment with picrotoxin suppressed the muscimol-induced feeding response. Error bars ± SEM; n = 11; mean values are shown at the bottom of each bar. (**a**) ** indicates significant differences compared to the aCSF group at matched times at *p* < 0.01. Δ indicates significant differences compared to the aCSF + muscimol group at matched times at *p* < 0.05. (**b**) Picrotoxin suppressed 24-h food intake by one-way repeated measures ANOVA, showing a significant combined effect of dose. ** indicates significant main effects at *p* < 0.01.

Next, we tested whether pretreatment with the GABA_A_ receptor antagonist, picrotoxin, would decrease muscimol-elicited feeding (Figure 4). Two-way repeated-measures ANOVA (n = 11) showed that both dose (F_(1.8, 9)_ = 8.67, *p* < 0.01, ${\eta_{p}}^{2}$ = 0.46) and time (F_(2, 9)_ = 24.93, *p* < 0.001, ${\eta_{p}}^{2}$ = 0.71) variables were significantly different, however, their interaction was not significant. Post hoc analysis revealed that the muscimol dose was significantly different from the second and the third picrotoxin doses, and all three-time points were significantly different from each other. In addition, post hoc analysis showed that the muscimol dose significantly increased food intake compared to the aCSF control condition. These data reveal that muscimol elicited significant feeding that was blocked by picrotoxin pretreatment (Figure 4a). More specifically, we suggest that picrotoxin decreased the action of muscimol at GABA_A_ receptors, and this action blocked the elicited feeding response. One-way repeated measures ANOVA of 24 h food intake showed that picrotoxin produced significant suppression (F_(4, 9)_ = 3.86, p = 0.01, ${\eta_{p}}^{2}$ = 0.28; Figure 4b). Water intake was not influenced by any dose of picrotoxin (Table S4), which further supports GABA_A_ receptors in the LS to be specifically involved in feeding and not drinking. These results provide converging evidence that muscimol elicited feeding by specifically activating GABA_A_ receptors.

### 3.5. Experiment 4: Suppression of LS GABA_B_ Receptors Reduced Agonist-Elicited Feeding

**Figure 5:**
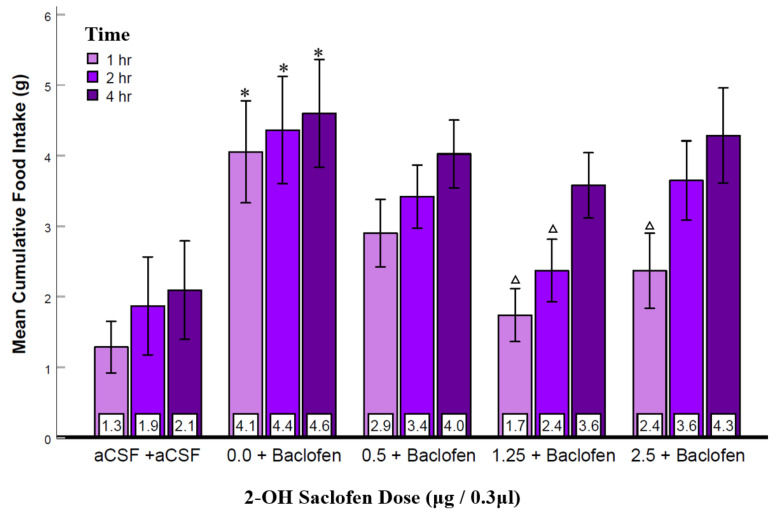
2-OH saclofen injected into the LS suppressed baclofen-elicited food intake. Error bars: ±SEM; n = 13; mean values are shown at the bottom of the bar. * indicates significant differences from the aCSF group at matched times at *p* < 0.05. Δ indicates significant differences compared to the baclofen and the 0.5 μg 2-OH saclofen groups at matched times at *p* < 0.05.

The fourth experiment examined whether pretreatment with the GABA_B_ receptor antagonist, 2-OH saclofen, would suppress the baclofen-elicited feeding. The intermediate dose of baclofen (1.125 μg) was used to prevent producing an effect that might be too large to suppress with the antagonist pretreatment. As shown in Figure 5, 2-OH saclofen decreased baclofen-elicited feeding. Although two-way repeated-measures ANOVA (n = 13) showed no significant effect of dose (F_(1.9, 11)_ = 2.37, *p* = 0.11, ${\eta_{p}}^{2}$ = 0.17) there was a statistically significant effect of time (F_(2, 11)_ = 23.7, *p* < 0.001, ${\eta_{p}}^{2}$ = 0.66), and importantly a dose and time interaction (F_(3.4, 11)_ = 2.91, *p* = 0.04, ${\eta_{p}}^{2}$ = 0.20). Justified by this significant interaction, post hoc analysis showed that baclofen, compared to the aCSF control condition, significantly increased eating at all three post-injection times. Importantly, saclofen suppressed this baclofen-elicited feeding in a variably dose-related manner that was most clearly manifest in the 1st and 2nd hours post-injection, with no statistically significant effects in the fourth hour (Figure 5). These data, demonstrating that higher doses of 2-OH saclofen produced significant suppression of baclofen-elicited feeding during the first 2 h, show that the suppressive effect of 2-OH saclofen was shorter and less pronounced than that of picrotoxin. One-way repeated measures ANOVA examining food intake at 24 h did not show significant effects (figure not shown). Water intake was also not significantly affected (Table S5). Together with the result of Experiment 3, these data provide further support for GABA_B_ receptor activation in the LS to be capable of eliciting feeding.

### 3.6. Experiment 5: Suppression of LS GABA_A_ Receptors at the Beginning of the Dark Phase Reduced Feeding

**Figure 6:**
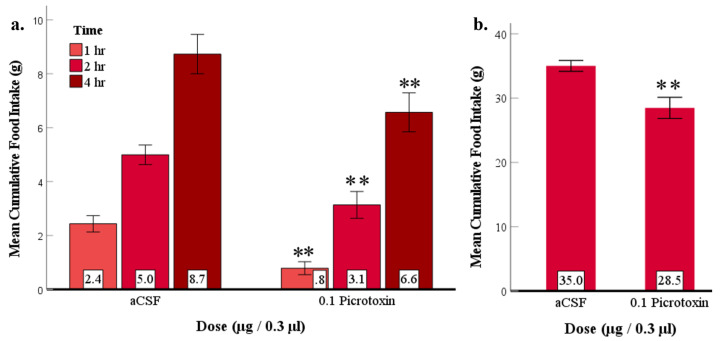
Picrotoxin injected into the LS at the beginning of the dark phase suppressed naturally occurring nocturnal feeding. Error bars: ±SEM; n = 11; mean values are shown at the bottom of the bar. ** indicates significant differences (*p* < 0.01) compared to the aCSF condition at matched times. (**a**) Picrotoxin supressed feeding at 1, 2, and 4 h after injection. (**b**) Picrotoxin continued to suppress feeding at the 24 h measurement.

In this experiment, the GABA_A_ receptor antagonist picrotoxin (0.1 μg) was injected into the rats’ LS within the first 30 min of the dark phase to explore whether GABA_A_ receptors are involved with naturally occurring feeding at the onset of the dark phase. The analysis showed that picrotoxin injection suppressed naturally occurring feeding (Figure 6). As a prelude to the core analysis, food intakes in the two aCSF tests (before and after picrotoxin tests) were compared by paired samples *t*-test, which revealed no significant differences. Therefore, the data from the two aCSF tests were combined to create an average for each subject. Similarly, two-way repeated measures ANOVA showed no significant differences between food intakes in the three picrotoxin injection tests; therefore, all three tests were combined. Two-way repeated measures ANOVA comparing mean aCSF and mean picrotoxin food intakes at 1, 2, and 4 h showed significant main effects for both dose (n = 11; F_(1, 9)_ = 13.38, *p* = 0.004, ${\eta_{p}}^{2}$ = 0.57) and time (F_(1.28, 9)_ = 72.15, *p* < 0.001, ${\eta_{p}}^{2}$ = 0.88); however, the interaction of drug and time was not significant (Figure 6a). Post hoc analysis revealed that picrotoxin significantly suppressed naturally occurring feeding at each post-injection time as compared to aCSF. Interestingly, one-way repeated measures ANOVA also showed a significant suppressive effect on 24 h food intake (F_(1, 9)_ = 21.58, *p* = 0.001, ${\eta_{p}}^{2}$ = 0.68; Figure 6b) suggesting that picrotoxin may have had anorexigenic effects that lasted for at least 24 h. Because picrotoxin suppressed feeding at the start of the rats’ active phase when they naturally eat, these results suggest that LS GABA_A_ receptors mediate naturally occurring nocturnal feeding. More specifically, we suggest that endogenous GABA activates LS GABA_A_ receptors at the beginning of the dark phase, inhibiting the GABA_A_ receptor expressing neurons, which results in feeding behavior.

### 3.7. Experiment 6: No Evidence That Suppression of LS GABA_B_ Receptors at the Beginning of the Dark Phase Reduced Feeding

**Figure 7:**
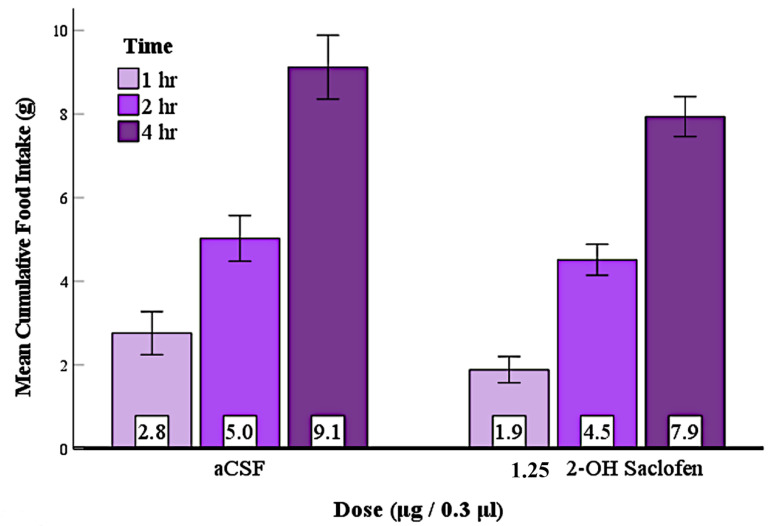
2-OH saclofen injection into the LS at the beginning of the dark phase did not significantly suppress natural feeding. Error bars: ±1 SEM; n = 12; mean values are shown at the bottom of the bar.

Next, we examined whether the GABA_B_ receptor antagonist 2-OH saclofen (1.25 μg) injected into the LS at the beginning of the dark phase would suppress natural feeding (Figure 7). Rats (n = 12) received an injection of the GABA_B_ antagonist 2-OH saclofen or the aCSF vehicle at the start of the dark phase. Food intakes between the two aCSF injection tests were compared by paired sample *t*-tests. These showed no significant differences; therefore, they were combined. Similarly, two-way repeated measures ANOVA comparing food intake after the three 2-OH saclofen injection tests showed no significant differences, and these groups were also combined. Two-way repeated measures ANOVA examining differences between the aCSF and the 2-OH saclofen groups’ food intakes after 1, 2, and 4 h showed no significant differences, indicating that 2-OH saclofen did not suppress food intake during the first 4 h of the dark phase (Figure 7). Moreover, one-way repeated-measures ANOVA of the 24 h food intake measurements showed no significant differences indicating that 2-OH saclofen had no delayed or long-term effects either. As the GABA_B_ antagonist did not significantly suppress naturally occurring nocturnal feeding, we have no evidence at this time indicating that GABA_B_ receptors are involved with the meal period associated with the beginning of the dark phase.

## 4. Discussion

Previous research has implicated the LS in feeding control mechanisms [4,11,17,24]. However, the types of receptors involved have received little attention. The current study had two aims: (1) to determine whether GABA_A_ and GABA_B_ receptor activation can elicit feeding in the LS, and (2) to explore whether endogenous GABA in the LS naturally activates GABA_A_ and GABA_B_ receptors during spontaneous feeding, especially that associated with the meals consumed by rats during the early dark-phase. This study duplicated the findings by Calderwood et al. [18,25], showing that muscimol in the LS elicited feeding, and we also showed that the effect was dose-dependent. Further, the present study shows that a GABA_A_ receptor antagonist can suppress this eating response. Other new findings are that eating is elicited by a GABA_B_ agonist in the LS, an effect reduced by a GABA_B_ antagonist. Most importantly, we show that the injection of a GABA_A_ antagonist can suppress feeding at the beginning of the dark phase. These findings collectively suggest that LS neurons expressing GABA_A_ and GABA_B_ receptors are elements of a neurocircuit that participates in the regulation of naturally occurring feeding.

To investigate GABA_A_ and GABA_B_ receptors in the feeding mechanism of the LS, we injected muscimol (Experiment 1) and baclofen (Experiment 2) to activate GABA_A_ and GABA_B_ receptors, respectively. Our results show that both agonists rapidly elicited dose-dependent feeding (Figure 2 and Figure 3), which aligns with previous research [24] showing that both muscimol and baclofen in the LS can stimulate sucrose intake. Since water intake was not significantly affected (Tables S2 and S3), this GABA receptor activation seems specific to eating and not drinking. It also indicates that the eating response was due to the agonists’ effect as a neurotransmitter on the receptors, and not due to a side effect, such as sedation. The average daily food intake at 24 h was also not significantly different from controls, suggesting that the feeding elicited by the GABA agonist injection was temporary and later compensated by less eating for the rest of the day. Our finding that baclofen was less potent in activating receptors than muscimol duplicates the results of previous research. Specifically, Mitra et al. [24] also found that GABA_A_ and GABA_B_ receptors had different effects on sucrose intake and concluded that perhaps GABA_B_ receptors stimulate sucrose licking and intake differently. The mechanisms underlying these apparent differences are unclear at this time. Regardless, both muscimol and baclofen injection in the LS resulted in a consistent and rapid increase in food intake. These results provide initial support for feeding induced by LS GABA_A_ and GABA_B_ receptor activation.

To provide further evidence for GABA_A_ and GABA_B_ receptor involvement, the antagonists picrotoxin (Experiment 3) and 2-OH-saclofen (Experiment 4) were injected into the LS before injecting their respective agonists. A new finding was that both antagonists suppressed the feeding elicited by their respective agonists (Figure 4 and Figure 5), providing converging evidence for both GABA_A_ and GABA_B_ receptors in LS stimulation of feeding behavior. Notably, picrotoxin injection blocked muscimol-elicited feeding in the initial 4 h post-injection and suppressed eating at least 24 h after the injection (Figure 4b). The 24 h suppression of feeding may have been due to a long-lasting inhibitory effect of picrotoxin on GABA_A_ receptors. Picrotoxin non-competitively blocks chloride channel activation [31] of GABA_A_ receptors, blocking endogenous GABA’s activation of those receptors. This property of picrotoxin may have contributed to its long-term effect. In alignment with our finding, LS injection of urocortin, a type of corticotropine-releasing factor, resulted in the suppression of feeding for 24 h [44] indicating the LS’s potential involvement in prolonged inhibition of eating. Another finding was that the high affinity GABA_B_ antagonist, 2-OH saclofen was less effective in suppressing food intake than the GABA_A_ receptor antagonist, picrotoxin and its effects were brief and not dose-dependent. It is possible that the injected 2-OH saclofen does not reach or bind to receptors with the same efficacy in the LS as in other brain regions. Nonetheless, the convergence of evidence from the agonists eliciting and antagonists suppressing feeding indicates that both GABA_A_ and GABA_B_ types of receptors in the LS can produce a robust feeding response.

Our second aim was to determine whether endogenous GABA might activate GABA receptors in the LS during the early dark phase when rodents’ food intakes are at a peak level. To investigate Aim 2, the first few hours of the dark phase served as a chronological marker for a time when rats are naturally motivated to eat. In our sample, the average cumulative food intake in the control group of naive rats for the first 4 h constituted 27% (10.8 ± 0.62 g) of their daily food intake (Supplementary Table S1). Similarly, Sindelar et al. [45] found that mice ate 26% of their total food intake within the same time frame. Several studies have shown that mammalian species have a homeostatic need to eat during this time because they do not eat much throughout the preceding inactive phase; consequently, when the dark phase starts, their energy stores are low [27,30,46]. Therefore, our study took advantage of this period to explore whether antagonizing LS GABA receptors may suppress naturally occurring feeding. To this end, we injected picrotoxin (Experiment 5) and 2-OH saclofen (Experiment 6) into the LS to determine this possibility. The data showed that picrotoxin strongly reduced naturally occurring feeding in the initial hours post injection (Figure 6a). As the antagonist presumably acts to prevent the activation of these receptors by endogenous LS GABA, a corollary conclusion is that the increase in natural feeding in the early dark phase is due—at least in part—to the activation of LS GABA receptors by the release of endogenous GABA. Additionally, we found a smaller anorexic effect was still evident 24 h after the initial injection of picrotoxin at the onset of the dark phase (Figure 6b). This result aligns with our previous finding showing a prolonged (over 24 h) effect of picrotoxin (Figure 4b) even when injected during the light phase. The immediate and prolonged suppression of feeding by picrotoxin may suggest that LS GABA_A_ receptor blockade produces effects that are not entirely compensated for by other mechanisms controlling food intake, thus highlighting the central role of this receptor.

On the other hand, 2-OH saclofen failed to significantly suppress naturally occurring feeding during the first 4 h of the dark phase even though it reduced baclofen-elicited feeding (Figure 7). It had no delayed effects since food intake after 2-OH saclofen injection was similar to controls even at 24 h. The current experiment does not provide support for a role for GABA_B_ receptors in naturally occurring feeding associated with the meal period at the beginning of the dark phase; however, this issue is unresolved and deserves further investigation. Future studies could investigate whether GABA_B_ receptors are involved with naturally occurring feeding using a different method or a different antagonist.

Given that GABA_A_ receptor activation is, with rare exceptions, inhibitory in mature mammals [20], the predicted impact of GABA on the LS neurons expressing GABA_A_ receptors is to inhibit their neural activity. Consequently, it would seem that GABA_A_ receptor activation in the LS elicits eating by inhibiting neurons that inhibit eating. Although the majority of the neurons in the LS are GABAergic [7], and we provide evidence that GABA_A_ and GABA_B_ receptors in the LS play a role in feeding behavior, one of the limitations of our study is that we do not know whether these GABA receptors are situated on the GABAergic neurons themselves or instead exert their effect through another neuronal type. In this regard, Sweeney and Yang [13] have opto/chemogenetic evidence that inhibition of GABAergic neurons originating in the septum and projecting to the lateral hypothalamus induces eating in mice. A parsimonious integration is that the LS GABA_A_ receptors we stimulated to elicit eating are expressed by the GABAergic LS neurons projecting to the lateral hypothalamus. This mechanism would align with our postulation that the action of GABA_A_ and GABA_B_ receptors in the LS is disinhibition through inactivating GABAergic neurons. Future studies may determine whether the LS GABA receptors mediating feeding are situated on GABAergic neurons.

The current study demonstrates that GABAergic receptor expressing neurons in this brain region may have a central inhibitory role in the feeding response, which is blocked when feeding is induced. This finding contributes to the current knowledge about the involvement of the LS brain region in the neural circuit of feeding. It may also have implications for studies investigating how these circuits relate to hunger, satiation, weight gain, or weight loss. In addition, this study may inform future research investigating the current obesity rates or eating and feeding disorders.

## 5. Conclusions

The current study was the first to investigate both GABA receptor types in the LS in control of eating behavior. Our results suggest that both GABA_A_ and GABA_B_ LS receptors participate in feeding control. The receptors can be activated to induce feeding and inactivated to suppress it. In particular, our study showed that GABA_A_ receptors are involved in naturally occurring feeding at the beginning of the dark phase. On the other hand, we had no evidence for GABA_B_ receptors to be involved in the same process. Nevertheless, our converging results provide evidence that either LS GABA_A_ or GABA_B_ receptor stimulation may be sufficient to produce feeding behavior and that LS GABA_A_ may be necessary for spontaneous natural feeding associated with the early hours of the dark phase. Since both the injected GABA agonists and the endogenous GABA in the LS suppressed GABAergic neurons and induced feeding, GABA receptors’ mechanism of action seems to be disinhibitory. This study contributes to our current understanding of the neural underpinnings of feeding and may contribute to the conception of theories relating to over- and under-eating. It also may have broader implications for understanding the increases in the rates of obesity and eating related disorders.

## Data Availability

The data presented in this study are openly available in Open Science Framework at doi:10.17605/OSF.IO/CVUNE.

## Supplementary Materials

The following are available at https://www.mdpi.com/article/10.3390/brainsci12070848/s1.

## References

[1] Hales C.M., Carroll M.D., Fryar C.D., Ogden C.L. (2020). Prevalence of Obesity and Severe Obesity among Adults: United States, 2017–2018. NCHS Data Brief, No. 360.

[2] Association A.P. (2022). Diagnostic and Statistical Manual of Mental Disorders, Text Revision (DSM-5-TR).

[3] Udo T., Grilo C.M. (2018). Prevalence and correlates of DSM-5–defined eating disorders in a nationally representative sample of US adults. Biol. Psychiatry.

[4] Azevedo E.P., Ivan V.J., Friedman J.M., Stern S.A. (2021). Higher-order inputs involved in appetite control. Biol. Psychiatry.

[5] Kosugi K., Yoshida K., Suzuki T., Kobayashi K., Yoshida K., Mimura M., Tanaka K.F. (2021). Activation of ventral CA1 hippocampal neurons projecting to the lateral septum during feeding. Hippocampus.

[6] Rizzi-Wise C.A., Wang D.V. (2021). Putting together pieces of the lateral septum: Multifaceted functions and its neural pathways. eNeuro.

[7] Sheehan T.P., Chambers R.A., Russell D.S. (2004). Regulation of affect by the lateral septum: Implications for neuropsychiatry. Brain Res. Rev..

[8] Olds J., Milner P. (1954). Positive reinforcement produced by electrical stimulation of septal area and other regions of rat brain. J. Comp. Physiol. Psychol..

[9] Pantazis C.B., Aston-Jones G. (2020). Lateral septum inhibition reduces motivation for cocaine: Reversal by diazepam. Addict. Biol..

[10] Altman J.L., Wishart T.B. (1971). Motivated feeding behavior elicited by electrical stimulation of the septum. Physiol. Behav..

[11] Urstadt K.R., Stanley B.G. (2015). Direct hypothalamic and indirect trans-pallidal, trans-thalamic, or trans-septal control of accumbens signaling and their roles in food intake. Front. Syst. Neurosci..

[12] Stanley B., Urstadt K., Charles J., Kee T. (2011). Glutamate and GABA in lateral hypothalamic mechanisms controlling food intake. Physiol. Behav..

[13] Sweeney P., Yang Y. (2016). An inhibitory septum to lateral hypothalamus circuit that suppresses feeding. J. Neurosci..

[14] Urstadt K.R., Kally P., Zaidi S.F., Stanley B.G. (2013). Ipsilateral feeding-specific circuits between the nucleus accumbens shell and the lateral hypothalamus: Regulation by glutamate and GABA receptor subtypes. Neuropharmacology.

[15] Zahm D.S., Parsley K.P., Schwartz Z.M., Cheng A.Y. (2013). On lateral septum-like characteristics of outputs from the accumbal hedonic “hotspot” of Peciña and Berridge with commentary on the transitional nature of basal forebrain “boundaries”. J. Comp. Neurol..

[16] Azevedo E.P., Tan B., Pomeranz L.E., Ivan V., Fetcho R., Schneeberger M., Doerig K.R., Liston C., Friedman J.M., Stern S.A. (2020). A limbic circuit selectively links active escape to food suppression. eLife.

[17] Terrill S.J., Jackson C.M., Greene H.E., Lilly N., Maske C.B., Vallejo S., Williams D.L. (2016). Role of lateral septum glucagon-like peptide 1 receptors in food intake. Am. J. Physiol. Regul. Integr. Comp. Physiol..

[18] Calderwood M.T., Tseng A., Stanley B.G. (2020). Lateral septum mu opioid receptors in stimulation of feeding. Brain Res..

[19] Stanley B.G., Lanthier D., Leibowitz S.F. (1988). Multiple brain sites sensitive to feeding stimulation by opioid agonists: A cannula-mapping study. Pharmacol. Biochem. Behav..

[20] Pierobon P. (2021). An interesting molecule:*γ*-aminobutyric acid. What can we learn from Hydra Polyps?. Brain Sci..

[21] Köhler C., Chan-Palay V. (1983). Distribution of gamma aminobutyric acid containing neurons and terminals in the septal area. Anat. Embryol..

[22] Xu Y., Lu Y., Cassidy R.M., Mangieri L.R., Zhu C., Huang X., Jiang Z., Justice N.J., Xu Y., Arenkiel B.R. (2019). Identification of a neurocircuit underlying regulation of feeding by stress-related emotional responses. Nat. Commun..

[23] Sweeney P., Yang Y. (2015). An excitatory ventral hippocampus to lateral septum circuit that suppresses feeding. Nat. Commun..

[24] Mitra A., Lenglos C., Timofeeva E. (2014). Activation of GABAA and GABAB receptors in the lateral septum increases sucrose intake by differential stimulation of sucrose licking activity. Behav. Brain Res..

[25] Calderwood M.T., Tseng A., Gabriella I., Stanley B.G. (2022). Feeding behavior elicited by mu opioid and GABA receptor activation in the lateral septum. Pharmacol. Biochem. Behav..

[26] Calderwood M.T. (2020). The Role of Lateral Septal Opioid and GABAA Receptors in Feeding Behavior. Doctoral Dissertation.

[27] Kersten A., Strubbe J.H., Spiteri N.J. (1980). Meal patterning of rats with changes in day length and food availability. Physiol. Behav..

[28] National Research Council (US) Committee for the Update of the Guide for the Care and Use of Laboratory Animals (2010). Guide for the Care and Use of Laboratory Animals.

[29] Percie du Sert N., Hurst V., Ahluwalia A., Alam S., Avey M.T., Baker M., Browne W.J., Clark A., Cuthill I.C., Dirnagl U. (2020). The ARRIVE guidelines 2.0: Updated guidelines for reporting animal research. J. Cereb. Blood Flow Metab..

[30] Page A.J., Christie S., Symonds E., Li H. (2020). Circadian regulation of appetite and time restricted feeding. Physiol. Behav..

[31] Sieghart W. (2006). Structure, pharmacology, and function of GABAA receptor subtypes. Adv. Pharmacol..

[32] Johnston G.A. (2014). Muscimol as an ionotropic GABA receptor agonist. Neurochem. Res..

[33] Bitran D., Dugan M., Renda P., Ellis R., Foley M. (1999). Anxiolytic effects of the neuroactive steroid pregnanolone (3*α*-OH-5*β*-pregnan-20-one) after microinjection in the dorsal hippocampus and lateral septum. Brain Res..

[34] Estrada-Camarena E., Contreras C.M., Saavedra M., Luna-Baltazar I., López-Rubalcava C. (2002). Participation of the lateral septal nuclei (LSN) in the antidepressant-like actions of progesterone in the forced swimming test (FST). Behav. Brain Res..

[35] Izquierdo I., da Cunha C., Rosat R., Jerusalinsky D., Ferreira M.B.C., Medina J.H. (1992). Neurotransmitter receptors involved in post-training memory processing by the amygdala, medial septum, and hippocampus of the rat. Behav. Neural Biol..

[36] Richter J.A., Gormley J.M. (1986). Inhibition of high-affinity choline uptake in the rat hippocampus by in vivo injection of phenobarbital in the medial septum. J. Pharmacol. Exp. Ther..

[37] Froestl W. (2010). Chemistry and pharmacology of GABAB receptor ligands. Advances in Pharmacology.

[38] Urwyler S., Gjoni T., Koljatić J., Dupuis D.S. (2005). Mechanisms of allosteric modulation at GABAB receptors by CGP7930 and GS39783: Effects on affinities and efficacies of orthosteric ligands with distinct intrinsic properties. Neuropharmacology.

[39] Paxinos G., Watson C. (2013). The Rat Brain in Stereotaxic Coordinates.

[40] Mauchly J.W. (1940). Significance test for sphericity of a normal n-variate distribution. Ann. Math. Stat..

[41] Greenhouse S.W., Geisser S. (1959). On methods in the analysis of profile data. Psychometrika.

[42] Bonferroni C. (1936). Teoria statistica delle classi e calcolo delle probabilita. Pubblicazioni del R Istituto Superiore di Scienze Economiche e Commericiali di Firenze.

[43] Gabriella I. (2022). Dataset of lateral septum—GABA receptors feeding study. Open Sci. Framew..

[44] Wang C., Kotz C.M. (2002). Urocortin in the lateral septal area modulates feeding induced by orexin A in the lateral hypothalamus. Am. J. Physiol. Regul. Integr. Comp. Physiol..

[45] Sindelar D.K., Palmiter R.D., Woods S.C., Schwartz M.W. (2005). Attenuated feeding responses to circadian and palatability cues in mice lacking neuropeptide Y. Peptides.

[46] Strubbe J.H., van Dijk G. (2002). The temporal organization of ingestive behaviour and its interaction with regulation of energy balance. Neurosci. Biobehav. Rev..

