# Supplementary material for: Stimulation of GABA Receptors in the Lateral Septum Rapidly Elicits Food Intake and Mediates Natural Feeding
Source: Brain Sci. 2022 Jun 28;12(7):848. doi: 10.3390/brainsci12070848 (PMC9312437; doi:10.3390/brainsci12070848)
Supplement: Supplementary file 1 [file brainsci-12-00848-s001.zip › brainsci-1756732-supplementary.pdf]

a. Experiment 1 Schedule:

Day 1: aCSF → Day 3: Muscimol → Day 5: Muscimol → Day 7: Muscimol

b. Experiment 2 Schedule:

Day 1: aCSF → Day 3: Baclofen → Day 5: Baclofen → Day 7: Baclofen

c. Experiment 3 Schedule:

Day 1: aCSF → Day 3: Muscimol → Day 5: Picrotoxin +Muscimol → Day 8: Picrotoxin +Muscimol → Day 11: Picrotoxin +Muscimol

d. Experiment 4 Schedule:

Day 1: aCSF → Day 3: Baclofen → Day 5: 2-OH saclofen + Baclofen → Day 8: 2-OH saclofen + Baclofen → Day 11: 2-OH saclofen + Baclofen

e. Experiment 5 Schedule:

Day 1: pre-aCSF → Day 3: Picrotoxin → Day 5: Picrotoxin → Day 7: Picrotoxin → Day 9: post-aCSF

f. Experiment 6 Schedule:

Day 1: pre-aCSF → Day 3: 2-OH saclofen → Day 5: 2-OH saclofen → Day 7: 2-OH saclofen → Day 9: post-aCSF

Figure S1: Experiment schedule. **a**. and **b**. Experiment 1 and 2, vehicle injections were on Day 1, and drug doses were in counterbalanced order. Rats were injected every 48 hours. **c**. and **d**. Experiments 3 and 4, schedule of injections of drugs and vehicle. The first two injections were 48 hours apart. After the second injection, rats received two injections, one after another every 72 hours. **e**. and **f.** Experiment 5 and 6, schedule of injections of drug and vehicle. Rats were injected every 48 hours.

Table S1. Mean baseline food intake during the light and dark phases. Spontaneous feeding was measured during the first 4 hours of the light phase (experiments 1 - 4) and the dark phase (experiments 5 - 6) in 2 groups of randomly selected rats.

	Time							
	1 hr		2 hrs		4 hrs		24 hrs	
	M	SEM	M	SEM	M	SEM	M	SEM
Light phase (n = 25)	1.57	0.31	2.24	0.39	3.8	0.57	39.72	1.3
Dark phase (n = 25)	3.66	0.38	6.61	0.48	10.8	0.62	39.51	1.12

Table S2. Mean water intake as a function of dose and time – Experiment 1: Muscimol did not affect water intake (n = 15)

Dose muscimol (µg/0.3µl)	Time 1hr	2hr	4hr	24hr
aCSF	2.1	3.0	4.1	30.1
0.1	2.3	2.7	5.4	36.1
0.2	1.5	2.8	4.3	34.3
0.3	1.7	2.4	4.1	34.2

Table S3. Mean water intake as a function of dose and time – Experiment 2: Baclofen did not affect water intake (n = 13)

Dose	Time			
baclofen (μg/0.3μl)	1hr	2hr	4hr	24hr
aCSF	2.5	3.6	4.7	30.3
0.45	1.9	3.0	4.3	29.8
1.125	1.8	3.0	4.2	32.7
2.25	1.9	2.7	5.0	30.6

Table S4. Mean water intake as a function of dose and time – Experiment 3: Picrotoxin did not affect water intake (n = 11)

Dose	Time			
picrotoxin (μg/0.3μl)	1hr	2hr	4hr	24hr
aCSF	1.8	2.5	2.8	30.5
0.0 + muscimol	2.2	3.9	4.9	33.4
0.05+ muscimol	3.0	3.9	6.2	38.4
0.1 + muscimol	3.7	4.9	6.4	32.0
0.2 + muscimol	3.4	4.9	6.3	34.0

Table S5. Mean water intake as a function of dose and time – Experiment 4: 2-OH saclofen did not affect water intake (n = 13)

Dose	Time			
2-OH saclofen (μg/0.3μl)	1hr	2hr	4hr	24hr
aCSF	2.1	2.9	3.7	25.4
0.0 + baclofen	2.1	3.4	5.2	28.3
0.5 + baclofen	2.5	4.2	5.6	28.8
1.25 + baclofen	1.9	3.1	5.1	28.6
2.5 + baclofen	2.0	3.4	4.4	24.4
